# Does Expecting Matter? The Impact of Experimentally Established Expectations on Subsequent Memory Retrieval of Emotional Words

**DOI:** 10.3390/jintelligence11070130

**Published:** 2023-07-01

**Authors:** Yueyue Xiao, Aiqing Nie

**Affiliations:** 1 Department of Psychology and Behavioral Sciences, Zhejiang University, Hangzhou 310028, China; 2 Department of Psychology, College of Educational Sciences, Shanxi Normal University, Taiyuan 030031, China

**Keywords:** expectation, stimulus emotionality, item memory, source memory, experimentally established expectation

## Abstract

Previous studies have confirmed that different degrees of expectation, including the bipolarity of the expected and unexpected, as well as an intermediate level (no expectation), can affect memory. However, only a few investigations have manipulated expectation through experimentally established schema, with no consideration of how expectation impacts both item and source memory. Furthermore, stimulus emotionality may also impact memory. Therefore, we conducted a study to investigate the effects of three levels of expectation on item and source memory while considering the impact of stimulus emotionality. The experiment began with a phase dedicated to learning the rules. In the subsequent study phase, negative and neutral words were manipulated as expected, no expectation, and unexpected, based on these rules. This was followed by tasks focused on item and source memory. The study found that there was a “U-shape” relationship between expectation and item memory. Additionally, the study revealed the distinct impacts of expectation on item and source memory. When it came to item memory, both expected and unexpected words were better remembered than those with no expectations. In source memory, expected words showed memory inferiority for expectation-irrelevant source information, but an advantage for expectation-relevant source information. Stimulus emotionality modulated the effect of expectation on both item and source memory. Our findings provide behavioral evidence for the schema-linked interactions between medial prefrontal and medial temporal regions (SLIMM) theory, which proposes that congruent and incongruent events enhance memory through different brain regions. The different patterns between item and source memory also support dual-process models. Moreover, we speculate that processing events with varying levels of emotionality may undermine the impact of expectation, as implied by other neural investigations.

## 1. Introduction

Previous experience can shape behavior, including shaping the expectation for forthcoming events (Greve et al. 2019; Lin and Liang 2020; Witherby and Carpenter 2022). Expectation is a mental process that involves estimating whether a desired or unwanted event or behavior will occur at a certain place or time. Humans can generate an expectation by observing recurring events, allowing them to quickly respond to upcoming consequences, either to secure an advantage or to avoid potential harm. Expectations are typically formed based on experience, where one certain event follows another and is repeated several times (Kafkas and Montaldi 2018a). Expectations can influence the way we encode, store, and retrieve information (Frank et al. 2018; van Kesteren et al. 2012; Witherby and Carpenter 2022), which has inspired us to delve into the investigation of expectation and memory.

The expectations in question may be relevant to our pre-existing schema or may be generated by experimenters in the lab (i.e., experimentally established expectations). So far, a large number of researchers (as seen in Section 1.1, Section 1.2, Section 1.3 and Section 1.4) have found that our memory for expected and unexpected events operates differently, and the memory for context is also influenced by expectation. To our best knowledge, no research has been conducted on whether experimentally established expectations would impact the memory of events and their associated contexts differently, or whether stimulus emotionality would modulate the pattern of these tasks. We review the literature and describe our research purpose below.

### 1.1. The Pre-Existing Expectation on Episodic Memory: Mixed Findings

Expectations can be generated by various schemata in our minds. Events that conform to the schema are regarded as expected, whereas those inconsistent with the schema are considered unexpected. Among the studies investigating the impacts of schema-driven expectations on long-term memory, two types of expectations can be successfully activated: pre-existing expectations and experimentally established expectations (Brod and Shing 2019; Küppers and Bayen 2014; van Kesteren et al. 2012). One line of research focuses on how pre-existing expectation modulates subsequent memory. Typically, participants are instructed to recall the subject of action after perceiving a set of stereotype-related episodes, which can be either expected or unexpected. For instance, a priest helping a stranger may be seen as expected, whereas a violent mob giving food to a stray dog is often regarded as unexpected.

Pre-existing expectations can also be generated from prior knowledge. For example, in a professor’s office, a coffee mug is likely to be found (i.e., expected) whereas a toy car is less likely (i.e., unexpected). To date, the prior-knowledge-elicited expectation has gained great attention from researchers (Frank et al. 2018; Prull 2015; van Kesteren et al. 2013; Wynn et al. 2020). Within this literature, investigations have utilized verbal materials and semantic knowledge as the primary factors capable of eliciting expectations (Atienza et al. 2011; Bein et al. 2014). 

Research has investigated how prior knowledge may influence our memory for expected and unexpected events, usually through classical study-and-test tasks (e.g., Frank et al. 2018; Prull 2015; Wynn et al. 2020). In these tasks, materials are prepared as both schema-congruent and schema-incongruent to naturally elicit expectations once the to-be-remembered stimuli activate certain schema. Schema-congruent events conform to expectations, while schema-incongruent events are seen as unexpected. Participants learn sets of stimuli and are subsequently given either a pre-announced or an impromptu memory test. This design allows researchers to compare the test performance between expected and unexpected encoding situations.

Episodic memory, which is distinct from semantic memory that relies on pre-existing knowledge, is responsible for storing diverse events in our daily life (Elibol-Pekaslan and Sahin-Acar 2018; Li and Nie 2021; Wang 2018; Weidemann et al. 2019). Two subtypes of episodic memory, namely item memory and source memory, have been identified as dissociable. Item memory allows us to distinguish previously experienced items from novel items, while source memory enables us to retrieve the peripheral details and the contexts from which an event was originally acquired (Ehrenberg and Klauer 2005; Li and Nie 2021; Nie et al. 2019a; Nie et al. 2019b; Ye et al. 2019).

According to dual-process models, episodic memory relies on two independent processes: familiarity and recollection. Familiarity is more automatic and reflects only a sense of knowing, whereas recollection is more consciousness-consuming and requires effort to recall the details (Cooper et al. 2017; Leynes et al. 2017; Nie et al. 2019b; Ye et al. 2019). Item memory, particularly when tested through recognition tasks, requires both processes, but familiarity plays a more significant role because a feeling of knowing is often sufficient to identify items. On the other hand, source memory depends more on the recollection-based process (Kragel and Polyn 2016; Li and Nie 2021; Minor and Herzmann 2019; Ritchey et al. 2018). Previous research has demonstrated that the ability to recall items relies heavily on the recollection process compared to the item recognition task (Kragel and Polyn 2016; Nie et al. 2019a; Rajan and Bell 2015). Single-process models, however, suggest that recall only involves one process. The differentiation between item and source memory is based on the strength of memory or the familiarity of an item (Hayes et al. 2017; Slotnick and Dodson 2005).

For item memory, many studies have investigated the impact of expectations on subsequent retrieval performance (e.g., Lin and Liang 2020; van Kesteren et al. 2010, 2013). Among them, research that adopted pre-existing schema to trigger expectations usually observed a memory advantage for expected information over unexpected information (van Kesteren et al. 2010). The retention of schema-related events has been associated with the expedited consolidation process for expected information resulting from the facilitation of pre-existing schema (Packard et al. 2017; van Kesteren et al. 2013). However, other studies obtained a reversed pattern where expectation-violated events were better retrieved than expected events (e.g., Prull 2015).

The reason for such differences might be that the test tasks were different in these studies. For instance, Prull (2015) applied a unique test paradigm, the modified remember–know paradigm, as opposed to the typical study-and-test tasks. In Prull’s (2015) study, participants were instructed to respond with a “remember” if they could retrieve any detail about the witnessed object, or a “familiar” if they sensed recognition but lacked specific details in memory. This remember–know approach potentially unlocks key insights into the roles of familiarity and recollection processes in memory processes. A “remember” response represents the recollection process, whereas a “know” response represents the familiarity process (Hayes et al. 2017; Nie et al. 2021b; Prull 2015).

Regarding the investigations into the other subtype of episodic memory, source memory, the modulation of pre-existing expectation has also been explored (e.g., Atienza et al. 2011; Bayen and Kuhlmann 2011; Küppers and Bayen 2014; Süssenbach et al. 2016). The performance has been mixed, with some investigations revealing the incongruency effect in source memory (Ehrenberg and Klauer 2005; Süssenbach et al. 2016), and others revealing that congruent events hold relatively superior source memory compared to incongruent events (Atienza et al. 2011; Bayen and Kuhlmann 2011).

### 1.2. Experimentally Established Expectation in Memory Studies: Mixed Findings

In addition to the above-mentioned pre-existing expectations, experimentally established expectations may also influence memory. As not all incoming information has pre-arranged schema, people need to constantly create new rules and use them to shape their behaviors. Perhaps the new rules will eventually become a part of our stable knowledge, i.e., into our pre-existing schema. Therefore, it is imperative to investigate whether the newly implemented rules have a dissimilar effect on our memory compared to the pre-existing schema.

There are two approaches to generating expectations during experiments. The first approach is to establish a hierarchy-related rule, where high and low values are factitiously attributed to particular items. For example, in Brod et al.’s (2015) study, a rule-learning phase was conducted before the classical study-and-test tasks. During the rule-learning phase, participants were introduced to several novel rules regarding the comparative speed of cartoon vehicles. In the following study session, the competition results of each vehicle pair either aligned with the learned rules or violated them, followed by a memory test on the competition results. Comparable hierarchical rules were employed in another study where participants comprehended the relative prices of two objects (Greve et al. 2019).

The second type of rule is the relationship between two arbitrary stimuli, learned by presenting cues and targets in sequence. An example of a study using this rule was conducted by Kafkas and Montaldi (2018a). Their study consisted of three stages: rule learning, study, and test. During the rule-learning phase, natural objects always followed certain meaningless symbols, whereas manmade objects were preceded by other symbols. In the subsequent study phase, an expectation of the type of next object was elicited once a cue was presented, followed by a memory test on the objects. Similarly, Lin and Liang (2020) also adopted the sequential presentation of cue symbols with negative and neutral pictures to elicit experimentally established expectations.

Similar to the pre-existing expectation case, mixed patterns also occur in investigations of the impact of experimentally established expectations on item memory. For example, Brod et al. (2015) found inferior retention of unexpected competition results compared to expected competition. On the contrary, by semantically processing the items during the study, participants demonstrated superior memory retention of unexpected objects as compared to expected objects as in Kafkas and Montaldi (2018a) and Sweegers et al. (2015). Thus, the inconsistent findings might be related to the depth of the encoding task. If semantic processing is encouraged, then better memory performance could occur. These inconclusive results inspired us to further contribute to deepening the understanding of how expectations, especially those elicited by the experimentally established rules, affect item memory.

However, the influence of experimentally established schema on memory has not gained much attention. As a consequence, little evidence has been provided. The rationale for investigating experimentally established expectations is that the schemas in our brains are not always fixed, and we are capable of generating new rules for the world and forming new expectations as certain regularities occur. As a result, experimentally established expectations enable us to shed light on how a newly generated schema could affect our memory. Therefore, we investigated how expectations modulated the retention of events by establishing a set of rules during the experiment.

Additionally, scarce evidence has been provided for the influence of expectation on source memory when it is triggered by a set of experimentally established rules. The only study, as far as we know, is that by Sweegers et al. (2015). Participants in Sweegers et al.’s (2015) study learned rules for arbitrary face–home associations before a study phase. The following tests included a source memory task, where a marked memory advantage for unexpected events over expected events was observed. This incongruency effect was consistent with the findings of some investigations that adopted a pre-existing schema in source memory (Ehrenberg and Klauer 2005; Süssenbach et al. 2016) but was inconsistent with the results of other studies (Atienza et al. 2011; Bayen and Kuhlmann 2011).

As there is only one study that has explored the experimentally established rules on source memory, it still cannot be determined whether the pattern relevant to such types of rules is stable or not. To find further evidence needed to comprehend the issue thoroughly, the current study intended to investigate the putative influence of expectation on source and item memory by establishing a set of experimental rules to elicit expectations. In addition, this study might also allow for an observation of the possible dissociation between item and source memory, and to differentiate whether dual-process models or single-process models are supported.

### 1.3. Beyond Expected and Unexpected: Another Possibility

For many events, there is no expectation (or unrelated) at all. For example, when someone is walking along the street, we perhaps never expect the person passing by to be a man or woman because both are equally possible.

Several investigations have acknowledged that merely comparing the episodic memory of expectation-congruent events with that of expectation-incongruent events is insufficient for the influence of expectation on memory (Bonasia et al. 2018; Frank et al. 2018; Greve et al. 2019). By manipulating an additional level of expectation, that of no expectation, studies observed the advantages of both expected and unexpected events over those of no expectation. This phenomenon has been described as a “U-shape” function of expectation by Greve et al. (2019). Such findings align with the schema-linked interactions between the medial prefrontal (mPFC) and medial temporal regions (MTL) (i.e., SLIMM) model proposed by van Kesteren et al. (2012). The model suggests that congruent and incongruent events can both enhance memory, albeit through different brain mechanisms. The mPFC is associated with processing information that is congruent with what we expect, whereas the MTL system functions to detect prediction errors (Bonasia et al. 2018; van Kesteren et al. 2012).

Despite the beneficial effect of having certain expectations, the extent to which the memory discriminability of expectation-congruent events is superior to expectation-incongruent events lacks consensus. Frank et al. (2018) reported a graded pattern, where congruent items had the best retention, incongruent items were in the middle, and the no-expectation item had the worst. However, this graded pattern was not observed by Bonasia et al. (2018), where expected and unexpected film clips were similarly recalled. The current study manipulated three levels of expectation—expected, no expectation, and unexpected—to provide support for the SLIMM model from the behavioral perspective. Furthermore, combining the purposes mentioned above, we aimed to determine whether the advantage of expected information exceeded that of unexpected information, in both item and source memory.

### 1.4. Stimulus Emotionality Modulates the Influence of Expectation on Episodic Memory

The regulatory function of *stimulus emotionality* on many cognitive processes, including episodic memory, has been investigated (e.g., Dunsmoor et al. 2019; Johnson and MacKay 2019; Madan et al. 2019; Nie and Jiang 2021; Wang 2018; Shukla et al. 2019). A consensus is that emotionally aroused events are better remembered than neutral events, termed the Emotionally Enhanced Memory (EEM) effect. Thus far, many studies concerning item memory have revealed reliable EEM effects (e.g., Minor and Herzmann 2019; Nie et al. 2021a; Santaniello et al. 2018; Ventura-Bort et al. 2020; Weymar et al. 2013). Theoretical accounts for the EEM effect suggest that emotional stimuli automatically capture more attention resources during encoding, leading to better storage with elaborative rehearsal and facilitating subsequent retrieval (Johnson and MacKay 2019). 

By contrast, the impact of stimulus emotionality on source memory is more complex. Some investigations have consistently found better memory for the contextual details of emotional stimuli than neutral stimuli, i.e., the EEM effect in source memory (Kensinger and Schacter 2006; Ventura-Bort et al. 2020; Yick et al. 2015). However, other studies have revealed different patterns. Some researchers have observed significant differences in source memory performance between emotional and neutral stimuli (Davidson et al. 2006; Minor and Herzmann 2019; Wang and Fu 2011). However, others detected that the emotional content of a stimulus could impair the retrieval of contextual details (Ferré et al. 2019; Mackenzie et al. 2015; Pereira et al. 2019).

The priority-binding theory states that when items and their contexts can be easily bound together, they are encoded simultaneously, allowing for the advantage of remembering contextual information in emotional stimuli to emerge. This reveals a reliable EEM effect in source memory (Ventura-Bort et al. 2020). However, if the binding between an item and its contextual information is resource-consuming, then the source memory of the emotional stimuli tends to be inhibited, as is reinforced by the attention-narrowing theory (Ferré et al. 2019). There are two subtypes of sources: intrinsic and extrinsic sources. Intrinsic sources are originally integral parts of the events (i.e., within-item features), whereas extrinsic sources are the features that are independent of the events. Intrinsic sources are easier to bind to the events than extrinsic sources (Mao et al. 2015; Pereira et al. 2019; Zhou et al. 2020), consistent with the priority-binding theory. 

In reviewing the literature on the impact of experimentally established expectations on episodic memory, there is only one recent study by Lin and Liang (2020) that examines the role of stimulus emotionality. Their investigation established an association of cue symbols (“%” or “#”) with pictures of certain valences (negative or neutral). Participants were presented with a series of cues and pictures in succession, where pictures were either expected or unexpected. The ensuing surprise recognition test revealed an interaction between expectation and stimulus emotionality. For the expected condition, memory for negative and neutral pictures was similar, while for the unexpected condition, stimulus emotionality hampered the storage of negative pictures as compared with neutral pictures.

Although the study conducted by Lin and Liang (2020) is innovative, it only explores how expectation affects item memory for stimuli of different emotionality, without addressing source memory. Furthermore, the third condition of expectation, where there is no expectation, has yet to be investigated. Therefore, our study aimed to explore the impact of stimulus emotionality on the influence of experimentally established expectations, in both the context of item memory and source memory. This can address the theoretical issue of whether dual-process models or single-process models are reinforced. If there is a differentiation in contributions, then a better understanding can be provided for the dual-process models that differentiate these two memory subtypes. However, if the patterns between item and source memory are similar, then the single-process models can be reinforced. Another benefit of our study is its examination of whether the contribution of stimulus emotionality can be further differentiated between item and source memory. If the differentiation is not verified, then it will support the attention-narrowing theory. Otherwise, the priority-binding theory will take effect. Notably, we also investigated the role of the level of no expectation, which has not been considered.

### 1.5. Predictions for the Current Study

In brief, the purpose of the current study was to investigate the impact of experimentally established expectations on item and source memory. Specifically, the study applied experimentally established rules. Previous studies primarily focused on the two ends of expectation, i.e., either expected or unexpected; however, in this study, we also manipulated the third condition of no expectation. Words of two emotional valences (negative and neutral) were adopted in the current study.

Inspired by the experimental procedures of Brod et al. (2015) and Kafkas and Montaldi (2018a), our experiment was composed of three phases: rule learning, study, and test. The rule-learning phase was designed for participants to discern the rules regarding the temporal sequences of cues and target words (i.e., whether they were negative or neutral), and to subsequently generate expectations or a lack thereof regarding the presentation of target words. During the study phase, words were displayed in response to the cues and manipulated to be expected, with no expectation, or unexpected, and were presented either in yellow or blue. A subsequent memory test was conducted, where participants were first instructed to take an item memory test distinguishing the studied words from new words. Then, participants were required to identify the displayed colors and preceding cues for the words they believed to be studied. These tasks were both source memory tasks, designated as color retrieval and cue identification, respectively. 

Additionally, after completing both the item memory and color retrieval tasks, participants were asked to rate their confidence in their responses. The confidence-rating task referred to the previous literature that intended both to explore the single- and dual-process models in memory and to reduce the impact of guesses (e.g., Blank et al. 2019; Greve et al. 2019; Pratte and Rouder 2011). The responses to the confidence-rating task and the task before were called “high-confidence responses” and “overall responses”, respectively.

From the above manipulations, we made the following predictions. First, for item memory, we predicted that both expected and unexpected words would be remembered better than words that were studied without an expectation (i.e., no expectation), in light of the SLIMM model and empirical results of several studies (Bonasia et al. 2018; Frank et al. 2018; Greve et al. 2019; van Kesteren et al. 2012). Further, we predicted that expected and unexpected events would be remembered at similar rates. Thus, a “U-shaped” pattern would be displayed, and this pattern would be similar in both the response sets of “overall responses” and “high-confidence responses”. In terms of stimulus emotionality, we predicted a standard EEM effect in item memory, consistent with several investigations that similarly used the Affective Norms for English Words (ANEW, Bradley and Lang 1999) as materials (Mneimne et al. 2010; Zhou et al. 2020). The EEM effect would support the theoretical accounts that emotional words can automatically capture more attention resources during the study phase, leading them to be better stored with elaborative rehearsal that also facilitates subsequent retrieval (Johnson and MacKay 2019).

Source memory might be divergent from item memory. For the source memory of color retrieval, we predicted that correctly retrieving the studied color for a word was equivalent to a “remember” response in the “remember–know” design and was related to the recollection process. On the other hand, an incorrect retrieval of the color case was akin to a “know” response and was related to the familiarity process. We expected to obtain an advantage for unexpected words compared with expected words, considering the results of Sweegers et al. (2015) and the last experiment by Greve et al. (2019). This prediction could also be supported by a substantial number of neural evidence that has found an association between detecting prediction errors and the activation of the MTL (e.g., Bonasia et al. 2018; Brod and Shing 2018; Kafkas and Montaldi 2018b; Greve et al. 2019). This can form a more recollection-based memory trace that is helpful to retrieve detailed information. If confirmed, our findings would provide support for the dual-process models rather than single-process models. The findings would indicate that the recollection process is largely involved in a source memory task that involves the retrieval of the color background of stimuli.

The task of cue identification, however, might show different patterns from that of color retrieval, because the cues were directly linked to the rules that elicited expectations. Consequently, a similar graded pattern, with the highest memory performance for expected words, was predicted, similar to that observed by Frank et al. (2018). Such hypothesized differences between color source and cue identification were reasonable because the colors and cues manipulated in the current study were different sources—intrinsic and extrinsic sources, respectively. Therefore, the recollection process’s contribution in these two tasks was different, with less involvement in color retrieval task and more in the cue retrieval task. Furthermore, no advantage for emotional words in source memory was expected, as the emotionality was added to the items themselves, but source information was non-emotional. A differential contribution of stimulus emotionality between item and source memory would support the attention-narrowing theory rather than the priority-binding theory.

Finally, we hypothesized that the stimulus emotionality would interact with the expectation in both item and source memory. Neural evidence has shown that memory for positive and neutral information is related to frontal activity, whereas negative information activates more temporal areas (Mickley and Kensinger 2009). Based on the SLIMM theory, these brain regions are related to the activity of processing expected and unexpected information, respectively. From this, we assumed that the contribution of expectation would interact with that of stimulus emotionality. Specifically, for negative words, memory enhancement or reduction might disappear or weaken in the condition of unexpected, whereas for neutral words, the same would be applied to the expected condition. Therefore, the attention-narrowing theory would apply to negative words in the unexpected condition and to neutral words in the expected condition.

## 2. Method

### 2.1. Participants

A total of 35 undergraduates and graduates (16 males and 19 females) were recruited to participate in the current experiment, and their ages ranged from 18 to 25 years old (*M* = 19.46, *SD* = 1.27). All of them were Chinese native speakers and were right-handed as determined by the Edinburgh Handedness Questionnaire, which was verified with a Cronbach α reliability of 0.869 among Chinese participants (Yang et al. 2018). All participants reported having normal visual acuity or had their vision corrected to normal, and none of them had color blindness. Additionally, they claimed to have no history or current symptoms of severe physical or neurological disorders. Before the experiment, the informed consent procedure was thoroughly explained to all participants, and written consent was obtained from each of them. Participants were given course credits or cash compensation for their participation. All experimental protocols adhered to the ethical standards outlined in the Declaration of Helsinki and were approved by the university’s Research Ethics Committee. After completing the experiment tasks, participants were debriefed and thanked by the experimenters.

After collecting all the data, five participants were excluded from further analyses. Two of them failed to complete the entire experiment due to technical issues, while the performance of another three participants exceeded three standard deviations (*SD*s) from the means in some of the conditions of either item or source memory. These have been considered to be extreme values under the assumption of data normality and have been rejected from analyses in many investigations (Flegal et al. 2010; Lange et al. 2019). Consequently, the valid sample size in our current experiment was 30.

Sample size estimation was conducted in the Software G*Power 3.1 (Faul et al. 2009). To achieve a small-to-medium effect size of 0.21 using the standard criteria (two-tailed α = 0.05, 1 − β = 0.80), for a repeated-measures analysis of variance (ANOVA) of within-subject factors, a reasonable number of participants in the sample pool should be 26. Furthermore, Greve et al. (2019) conducted a study with a sample size of 24 participants and were able to establish the presence of congruency and incongruency effects. This study resulted in 85% power to detect these effects. Similarly, Lin and Liang (2020) studied emotional valence by incongruence with a sample size of 29. Therefore, our current sample size not only met the estimation requirement but also exceeded that of previous empirical studies.

### 2.2. Design

The experimental design was a 3 (expectation: expected vs. no expectation vs. unexpected) × 2 (stimulus emotionality: negative vs. neutral) within-subject design. Expectation was manipulated by both cues and rules. Participants perceived the words following the “with rule” cues with expectations according to the rules, and those that aligned with the cue target rules were seen as expected, otherwise unexpected, whereas the words that followed the “no rule” cue were regarded as no-expectation words. See the “Section 2.3” Section for details. Stimulus emotionality was the emotional valence of words. The tests comprised three tasks: item memory, color retrieval, and cue identification, with the latter two tasks measuring source memory. 

### 2.3. Materials

A set of 454 words, consisting of half negative and half neutral in emotional valence, was adopted as the stimuli in the current experiment. All of these words were extracted from the ANEW lexicon and were translated into two-character Chinese words. These affective words had also been employed in previous investigations (e.g., Ke et al. 2017; Nie et al. 2023; Nie and Jiang 2021; Zhang et al. 2018; Zhou et al. 2020). The emotional valence was rated 1.22–2.49 for negative words (*M* = 2.00, *SD* = 0.31) on the nine-point Likert scale. This rating score was lower than that of neutral words, which varied from 3.47 to 7.00 (*M* = 5.85, *SD* = 0.86), *t*(452) = −63.332, *p* < 0.001, Cohen’s *d* = −5.945. It is worth noting that the arousal level was, even though we tried to control it, still higher for negative words (*M* = 6.35, *SD* = 0.97) compared with neutral words (*M* = 5.93, *SD* = 0.56), *t*(452) = 5.564, *p* < 0.001, Cohen’s *d* = 0.522. This difference in valence was caused by a limited pool of affective words, particularly due to insufficient available words in the Chinese norm. A considerable number of words were omitted from the current study due to cultural differences and linguistic characteristics. For instance, some words that are considered neutral in Western countries are viewed negatively in Chinese, while some words containing less than or more than two characters were disregarded.

The words were categorized into three groups. The first group, which consisted of 18 words, comprising 9 negative and 9 neutral, was assigned to the rule-learning phase. The goal was to assist participants in understanding and identifying the rules involved. Second, 420 words were used throughout the study and test phases. To avoid overwhelming participants and to minimize interference among stimuli, the words were separated into 10 blocks. A pilot experiment found that if only one or a few blocks were used, then the performance in both item and source memory tests would be lower than the chance level, making the outcomes challenging to imperfect. Therefore, we decided to use 10 blocks, each containing 42 words. Within each block, 30 words were designated as targets during the study phase. These targets were presented in a counterbalanced manner in terms of expectation and stimulus emotionality.

Additionally, two fillers were included in each block—one presented at the very beginning and the other presented at the end of the study block. All of the target words from the study phase, along with 10 new words (5 negative and 5 neutral), were subsequently tested in each block. The number of new words was lower because they did not have any experimentally elicited expectation, unlike the old words. Finally, the remaining 16 words (8 negative and 8 neutral) were used to practice the study-and-test method to ensure a comprehensive understanding of the experimental procedure. The procedure of the entire experiment is clearly illustrated in Section 2.4.

**Figure 1:**
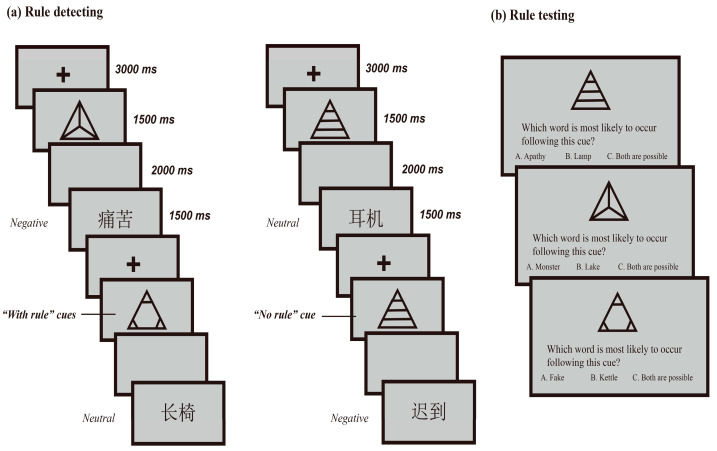
Schematic illustration of the procedures of rule learning. (**a**) Rule-detecting phase, where participants were instructed to view the display of cue and target words and learn the relationship between them. (**b**) Rule-testing phase, which was conducted to test whether the rules were fully learned. The meaning of “痛苦”, “长椅”, “耳机”, “迟到” are “pain”,” bench”,” earphone”,” late” respectively.

**Figure 2:**
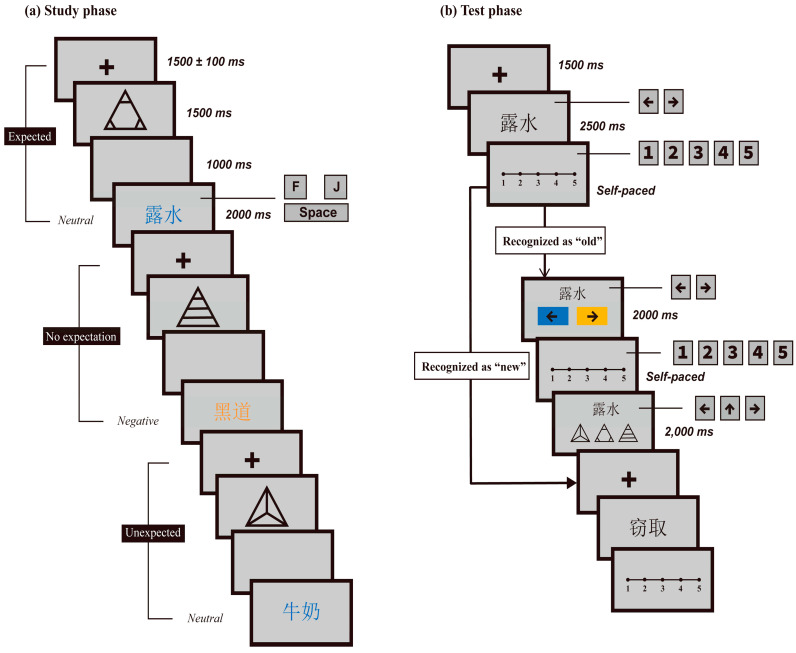
Schematic illustration of the procedures of the study and test phases. (**a**) The study phase, where participants completed the task in which words could be manipulated as either expected, no expectation, or unexpected. (**b**) The procedure of test tasks included item and source memory for colors and cues, followed by retrospective confidence judgments. The meaning of “露水”, “黑道”, “牛奶”, “窃取” are “dew”,” underworld”,” milk”,”stealing” respectively.

In addition to the words, three line drawings of symbols were created and served as cues. The symbols were all triangles in shape, of the same size (460 × 460 pixels), but had distinct patterns. See Figure 1 and Figure 2 for these triangles. Two of the shapes were defined as “with rule” cues, associated with certain rules in the rule-learning phase. Words following these cues were constantly negative or neutral, similar to the investigations of Kafkas and Montaldi (2018a) and Lin and Liang (2020). However, following another symbol, negative and neutral words were randomly displayed. Therefore, this symbol served as the “no rule” cue. The symbols were utilized in the study phase to assist participants in generating or not generating an expectation. Subsequently, words that came after the “with rule” cues could be deliberately categorized as either expected or unexpected, while those that followed the “no rule” cue were considered as having no expectation.

### 2.4. Procedure

The current study lasted approximately two hours and consisted of three phases: rule learning, study, and test. Upon arriving at the experimental lab, participants were seated in an individual room with no interruptions from others. They were positioned approximately 60 cm away from the monitor and instructed to keep their eye level at the same height as the center of the monitor. The entire experiment was programmed using MATLAB 2019b, and all the stimuli were presented against a light-grey screen, with a resolution of 1920 × 1080 pixels and a refresh rate of 100 Hz.

At the start of the experiment, participants were asked to complete a rule-learning task. This task was only given once. Therefore, before this task, participants were informed about the significance of the task and were told that the rules they learned would be frequently applied to the subsequent study-and-test tasks. Each block consisted of study and test phases, separated by a one-minute distractor task. The interval between blocks was 4 ± 1 min, as determined by participants. That is to say, after a minimum of three minutes of rest, they could start the next block if they felt ready, or they could rest for five minutes at most. Each participant was given practice trials before engaging in the formal 10 blocks of study-and-test tasks. These trials aimed to ensure that the participants could establish a clear understanding of how to apply the relevant rules to the study task, as well as how to complete the following tests. All protocols were identical between practice and the formal experiment. The following sections illustrate the details of each phase.

#### 2.4.1. Rule Learning

To establish a new set of schemas that can evoke specific expectations in the laboratory environment, a rule-learning phase was used. This phase was divided into two sessions: rule detecting and rule testing. The schematic illustration for the rule-learning process can be found in Figure 1.

During rule detecting, a series of cues (represented by three symbols) and targets (comprising two types of words, i.e., negative and neutral) were displayed on the screen in sequence. Participants were required to identify and comprehend the relationships between the cues and targets. Amongst the trio of cues, two of them (known as the “with rule” cues) were consistently linked with either negative or neutral words, whereas the third cue (referred to as the “no rule” cue) was randomly associated with both possibilities. Participants were made aware that it was possible to perceive an anomaly in one of the cue symbols. In Greve et al.’s (2019) study, participants could fully understand a rule across four trials. In our current experiment, six trials were used for each cue type, i.e., six trials for cue 1 with negative words, six trials for cue 2 with neutral words, and six trials for cue 3 with random words. The symbols that served as cues were always the same for each participant, but they were counterbalanced across participants.

Each trial began with a fixation cross, indicated by a “+” symbol, displayed in the screen center for 3000 ms, followed by a presentation of one of the three symbols for 1500 ms. Then, a grey, blank screen was shown for 2000 ms, which was followed by the target word displayed for a duration of 1500 ms. To prevent participants from relating the cues with other perceptual factors of the words, the target words were all displayed in black using the font of Microsoft Yahei and sized 192 pixels. In this session on oral communication, it was found that participants were aware of the nature of the words whether they were negative or neutral. This finding is consistent with previous studies by Kafkas and Montaldi (2018a) and Lin and Liang (2020).

Then, participants were instructed to complete a quiz on the rules they had discovered. A set of 3-alternative-forced-choice (3-AFC) questions was shown on the screen. On the top of the screen depicted a cue symbol, with the question “Which word is most likely to occur following this cue?” in the middle, and the three choices below, one was a negative word, one was neutral, while the final option read, “Both are possible”. Participants were told to make a choice based on their actual findings in the rule-detecting session. The 3-AFC questions were self-paced and participants received feedback on correct/incorrect after their responses were made. Afterwards, participants were instructed to use the rules to complete the 10 study-and-test blocks described below.

#### 2.4.2. Study

During the study phase, cue symbols and target words were displayed in sequence, similar to the rule-learning phase. However, three conditions were manipulated: expected, no expectation, and unexpected. For the “with rule” cues, their corresponding words were regarded as expected if they accorded with the rule. Otherwise, the cues were seen as unexpected. For the “no rule” cue, its subsequently displayed words were still randomly negative or neutral; therefore, they were defined as having no expectation. Additionally, the words were displayed pseudo-randomly in yellow or blue. The font size, type, as well as screen background color of the words were identical to those used in the rule-learning phase.

Within each block of the trial, a fixation cross was first presented at the center of the monitor for 1500 ± 100 ms. This was followed by a cue symbol for 1500 ms, a blank screen for 1000 ms, and finally, the target word was displayed for 2000 ms. Participants were instructed to respond to the word within this timeframe, based on whether it was expected or not; by pressing press “F” if it was expected, “J” if it was unexpected, or the “Space” bar if they did not expect anything. Key pressing was performed regardless of the color of the displayed words. During the key press for the trials, the participants were informed to pay close attention to both the words and their colors as they would be subsequently tested on both aspects. Additionally, they were instructed to memorize the relationship between the words and their cues, because the cue state of the words would also be tested. The purpose of offering instructions to participants to pay close attention to both color and cue was to reduce their bias towards one of them.

The symbols representing the three levels of expectation, namely expected, no expectation, and unexpected, remained the same as those from the rule-learning stage for each participant. Participants used their index and thumb fingers to press the keys, and the assignments of the fingers were counterbalanced across participants. Moreover, the fingers utilized to press keys were balanced within different blocks for each participant. Participants were encouraged to respond as quickly and accurately as possible. Each block included 30 trials in a pseudo-random order to prevent three or more successive trials in an identical condition. The trials were balanced based on expectation, stimulus emotionality, and color, except for the two fillers that were always in the expected condition. This resulted in 10 expected, 10 no-expectation, and 10 unexpected trials in each block, half negative and half neutral in each condition. The schematic illustration for the study phase is displayed in Figure 2a.

#### 2.4.3. Distractor Task

Following each study phase of the block, there was a 1 min distractor task where participants had to answer some arithmetic questions. This was conducted before the subsequent test tasks to prevent any possible self-recitation or recall attempts. The questions, including multiple choices and blank fillings, were extracted from the Chinese college entrance exams and printed on paper. Participants were instructed to answer these questions as indicated on the screen. Meanwhile, the program counted down for 60 s before flashing to the welcome page of the test phase with a beep to inform participants. At this point, they were instructed to stop answering the arithmetic questions and to start the test tasks.

#### 2.4.4. Test

Within each block, all 30 target words in the study phase that were previously displayed were tested and mixed with another set of 10 new words. The lower number for new words was because they did not have any experimentally elicited expectation, whereas the old words had three levels of expected, no expectation, and unexpected. During the memory test trials, a fixation cross appeared on the screen for 1500 ms, followed by a black-colored display of the to-be-tested word for 2500 ms. Additionally, the font size, type, as well as screen background color of the words were consistent with the rule-learning phase.

The test tasks were designed following a sequential paradigm, where participants were required to retrieve the item at least twice. Firstly, they were to retrieve the item itself, and then recall its context(s) during the previous study phase. The sequential paradigm has been used in investigating source memory, and in distinguishing item memory from source memory (Li and Nie 2021; Nie et al. 2013; Nie et al. 2019a; Ventura-Bort et al. 2016; Yick and Wilding 2014). In our current study, we asked participants to first determine if the displayed word was studied or not. They were instructed to press the left arrow if it was new and the right arrow if old, and afterwards to rate their confidence in their response. The responses were made from 1 (“not sure at all”) to 5 (“sure”) by pressing 1, 2, 3, 4, or 5 on the keyboard. The confidence scale was represented by a horizontal line with five points, set from the left end to the right end, with numbers printed below (see Figure 2b). To reduce the influence of guessing responses in old judgments during memory tests, we incorporated a confidence-rating design. This approach is in alignment with that used in several previous studies (e.g., Blank et al. 2019; Greve et al. 2019; Pratte and Rouder 2011). 

Two additional tests were conducted on words that were categorized as old: color retrieval and cue identification. The first test involved retrieving the displayed colors of the old words, where participants were instructed to choose whether the currently displayed word (i.e., the one just classified as old) was previously displayed in yellow or blue by pressing the left arrow and right arrow on the keyboard, respectively. The word was presented at the top of the screen, with two rectangles identifying the color and the corresponding key below. Afterwards, participants completed another five-point confidence-rating scale on their responses.

The second test involved identifying the preceding cues of words. With the word still presented at the top, participants were requested to identify the cue symbol that was displayed before the target word in the previous study phase. They had to choose from three options and press the corresponding number on the keyboard. It is worth noting that for the cue-identification task, participants were told to choose the cue they saw in the study phase rather than a cue that followed the rule along with the to-be-tested word.

These two additional memory tests (color retrieval and cue identification) required the participants to respond to each trial within 2000 ms. When they failed to respond within the time limit, then a “timeout” screen would appear for 500 ms. The two confidence ratings were self-paced, and participants were instructed to answer truthfully. Additionally, the manipulations of finger assignments for key pressing, font size, word type, pseudo-random word order, background color, speed, and accuracy were similar to those used in the previous study phase. A schematic illustration of the test phase can be seen in Figure 2b.

## 3. Data Analysis and the Results

The data were analyzed using IBM SPSS Statistics (version 23). A significance level of *p* < 0.05 (two-tailed) was set for all inferential analyses. In cases where a sphericity violation occurred during repeated-measures ANOVAs, the Greenhouse–Geisser method was adopted to correct the degree of freedom (*df*). The *F* ratios, together with corrected *p*-values and effect size ($\eta_{p}^{2}$), were reported. Post hoc tests were conducted to examine significant effects using the Bonferroni method for multiple comparisons. Mean differences (MDs) and the 95% CIs (confidence intervals) were also reported. For *t*-tests, the *p*-values and effect sizes, as measured by Cohen’s d, were reported. Data of interest are reported below for each phase.

### 3.1. Accuracy of Rule Learning and Study

To verify the manipulation of the experimentally established expectations in the rule-learning and study phases, we compared the accuracy of judgments in these phases to the chance level. The mean accuracy of answering the rule-related questions in the rule-learning phase was 0.939 (*SD* = 0.127), which was significantly higher than the chance level of 0.333 for the 3-AFC question; *t*(29) = 26.161, *p* < 0.001, Cohen’s d = 7.365. These results show that participants had accurately detected the rules during rule learning, which allowed them to generate correct expectations in the subsequent study phase. During the study, prominently higher accuracy was also found (*M* = 0.918, *SD* = 0.076) when compared to the chance level of 0.333; *t*(29) = 43.430, *p* < 0.001, Cohen’s d = 12.304. This outcome not only indicates that the participants were applying the correct rules to generate the appropriate expectations (or not to have any expectations) but also suggests that our manipulation of the three conditions of expectation was successful. Further, the three levels of expectation did not differ from each other, with *p* ≥ 0.879. Thus, our current study task was not affected by expectation manipulation as indicated by the results.

### 3.2. Accuracies for Item Memory

**Figure 3:**
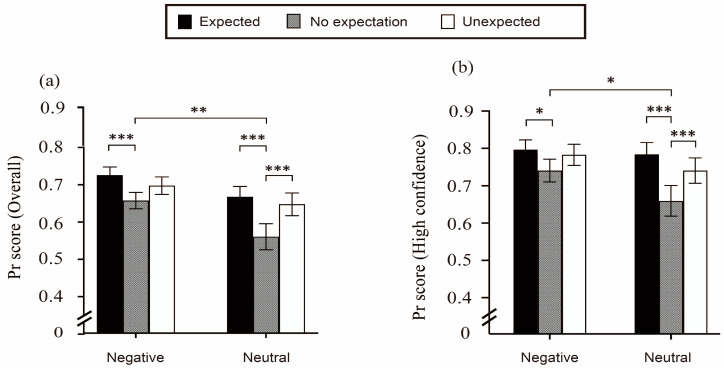
Performance in item memory task (represented by Pr scores (hit rates—false alarm rates) of the negative and neutral words in the expected, no-expectation, and unexpected conditions for (**a**) overall responses and (**b**) high-confidence responses). Bars represent the standard deviations (*SDs*). * *p* < 0.05, ** *p* < 0.01, *** *p* < 0.001.

As for the subsequent memory performance, the accuracy of item memory was first represented by the hit rates (hits) for each condition and was further considered to be indexed by the Pr scores (Prs). The Prs were calculated by the subtraction of the hit rates and the corresponding false alarm rates. The performance for item memory was also calculated and analyzed for those who responded with high confidence (i.e., rated 4 or 5 on the confidence scale). The indexes for both Prs and high-confidence responses were to mitigate the potential disturbance caused by guessing. In addition, the d’ for both overall responses and high-confidence responses was also calculated, which was the subtraction of the Z_hits_ from the corresponding Z_false alarms_. The overall hit rates, Prs, and d’ across the conditions in item memory are shown in Table 1, together with those of the high-confidence responses. The accuracies (indexed by Prs) of the overall responses and the high-confidence responses are illustrated in Figure 3. Because we had no prior predictions about the reaction times, we placed the data of the reaction times related to item memory in a file of Supplementary Materials.

Incidentally, the item memory results showed that the mean number of trials between “overall responses” and “high-confidence responses” were similar. Specifically, for the expected negative words, the trial number was 43, for the unexpected neutral words it was 42, for unexpected negative words it was 42, for expected neutral words it was 43, for negative words with no expectation it was 40, and for neutral words with no expectation it was 35. In the high-confidence responses, for the expected negative words, the mean trial number was 40, for the unexpected neutral words it was 37, for expected negative words it was 38, for expected neutral words it was 39, for negative words with no expectation it was 35, and for neutral words with no expectation it was 30.

For the accuracies, repeated-measures ANOVAs with expectation (expected, no expectation, and unexpected) and stimulus emotionality (negative and neutral) as the within-subject factors were conducted to explore how they contribute to item memory. The accuracies of the overall responses and high-confidence responses were submitted to the repeated-measures ANOVA of expectation and stimulus emotionality for the separate analyses. Before the ANOVA, we tested the sphericity. Mauchly’s test of sphericity for both effects of expectation and the interaction showed that the assumption of sphericity was not violated: for expectation, *χ*^2^(2) = 5.251, *p* = 0.072 (overall responses) and *χ*^2^(2) = 4.842, *p* = 0.089 (high-confidence responses); for interaction effect, *χ*^2^(2) = 2.779, *p* = 0.249 (overall responses) and *χ*^2^(2) = 3.094, *p* = 0.213 (high-confidence responses). Therefore, no Greenhouse–Geisser correction was applied. 

The ANOVA revealed a reliable main effect of expectation for both the overall responses (*F*(2,58) = 27.573, *p* < 0.001, $\eta_{p}^{2}$ = 0.487) and high-confidence responses (*F*(2,58) = 21.553, *p* < 0.001, $\eta_{p}^{2}$ = 0.426). The post hoc test found higher item accuracy for the targets with expectations than those without any expectations. Expected words were remembered better than no-expectation words; *t*(29) = 7.193, *p* < 0.001, and Cohen’s *d* = 1.313 for the overall responses; *t*(29) = 6.434, *p* < 0.001, and Cohen’s *d* = 1.175 for the high-confidence results. Furthermore, unexpected words also showed a memory advantage over the no-expectation words; *t*(29) = 5.196, *p* < 0.001, and Cohen’s *d* = 0.949 for the overall responses; *t*(29) = 4.348, *p* < 0.001, and Cohen’s *d* = 0.794 for the high-confidence responses. However, no difference was found between the expected and unexpected words for item memory; *t*(29) = 1.996, *p* = 0.152, and Cohen’s *d* = 0.364 for the overall responses; *t*(29) = 2.087, *p* = 0.124, and Cohen’s *d* = 0.381 for the high-confidence responses. 

This finding suggested that both expected and unexpected words had an advantage over the words with no expectation in terms of item memory, thus the relationship between expectation and item memory followed a “U-shaped” curve. This outcome suggested that the “U-shaped” curve regarding item memory was similar for the two sets of responses, namely “overall responses” and “high-confidence responses”. Such similarity is in line with that found by Greve et al. (2019). In summary, the main effect observed in item memory, for both the overall and high-confidence responses, confirmed our assumption that both expected and unexpected words have a memory advantage compared to information that was studied without an expectation. Additionally, our findings showed that both expected and unexpected events behaved similarly.

Meanwhile, there was also a significant effect of stimulus emotionality (*F*(1,29) = 12.319, *p* < 0.001, $\eta_{p}^{2}$ = 0.298 for overall responses; *F*(1,29) = 5.558, *p* < 0.001, $\eta_{p}^{2}$ = 0.161 for high-confidence results) where the negative words were much better remembered than the neutral words, indicating a reliable EEM effect. Importantly, there was also an interaction effect of expectation and stimulus emotionality across the overall responses (*F*(2,58) = 3.623, *p* = 0.033, and $\eta_{p}^{2}$ = 0.111) and in responses with high-confidence (*F*(2,58) = 4.095, *p* = 0.022, $\eta_{p}^{2}$ = 0.124). The significant interaction was further analyzed using several post hoc multiple comparisons. For the overall responses on item memory, as shown in Figure 3a, there was a standard “U-shape” of expectation for the neutral words, which was the pattern of both expected and unexpected words over the words of no expectation. This outcome demonstrated that the neutral words in the expected and unexpected conditions could be more accurately identified than those in the no-expectation condition; *t*(29) = 6.995, *p* < 0.001, MD = 0.107, and 95% CI [0.061, 0.153]; *t*(29) = 5.648, *p* < 0.001, MD = 0.087, and 95% CI [0.041, 0.133].

For the negative words, only an advantage of expected over no expectation was found; *t*(29) = 4.431, *p* < 0.001, MD = 0.0.68, and 95% CI [0.022, 0.114]. There were no differences between the expected and unexpected conditions for negative words (*t*(29) = 1.825, *p* = 0.167), nor for neutral words (*t*(29) = 1.347, *p* = 0.247). In addition, as for the effect of stimulus emotionality in the interaction, the standard EEM effect (better memory for negative than neutral words) was only found when there was no expectation for the target words; *t*(29) = 4.359, *p* = 0.001, MD = 0.097, and 95% CI [0.028, 0.166]. Furthermore, these data confirmed our previous discovery that the emotionality of a stimulus can play a role in the influence of item memory by expectation.

The interaction across conditions for high-confidence responses is evident in Figure 3b, which followed the same trend as the overall responses. The neutral words also showed a “U-shape” pattern of expectation, with both expected and unexpected words occurring more frequently than words with no expectation. Moreover, the accuracy for the expected words was higher than for words with no expectation (*t*(29) = 6.777, *p* < 0.001, MD = 0.106, 95% CI [0.059, 0.152]), and there was better item memory in the unexpected condition than in the no-expectation condition, *t*(29) = 4.387, *p* < 0.001, MD = 0.068, 95% CI [0.022, 0.115]. For negative words, the expected targets were more accurately remembered compared with their no-expectation counterparts; *t*(29) = 3.141, *p* = 0.032, MD = 0.049, and 95% CI [0.002, 0.096]. In addition, no significant divergence was found between the expected and unexpected conditions; *t*(29) = 0.827 and *p* = 0.872 for negative words; *t*(29) = 2.390 and *p* = 0.106 for neutral words. Similarly, significantly better item memory for negative words than neutral words was only elicited in the condition of no expectation (*t*(29) = 3.415, *p* = 0.018, MD = 0.068, 95% CI [0.007, 0.129]), demonstrating an EEM effect in the no-expectation circumstance. Overall, our hypothesis that the interaction between stimulus emotionality and expectation affects item memory was confirmed, particularly in the high-confidence responses.

The results of the two-way repeated-measures ANOVA for d’ in item memory indicated that there were no significant main effects of expectation or stimulus emotionality on the overall responses, with *p* ≥ 0.726. Additionally, the interaction between these factors was not statistically significant, with *p* = 0.999. Likewise, no main effects or interactions for either factor were observed in the high-confidence responses, with *p* ≥ 0.772. These findings suggest that neither the overall responses nor high-confidence responses in item memory were sensitive to the manipulated variables in d’.

**Figure 4:**
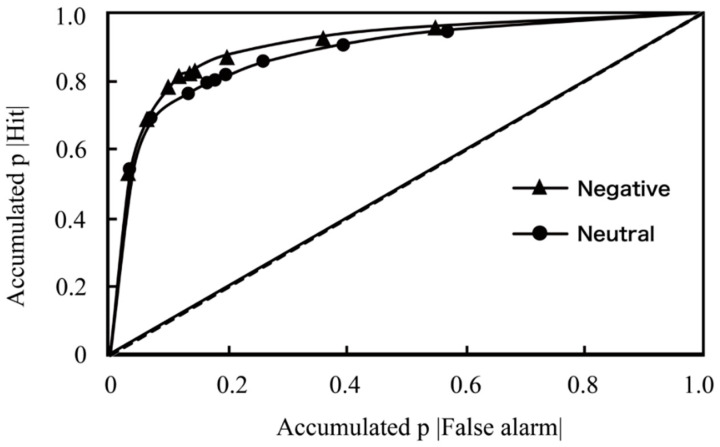
ROC curves in item memory task, plotting the cumulative hit rates and false alarm rates across confidence ratings.

Additionally, the receiver operating characteristic (ROC) curves were obtained separately for negative and neutral words in the item memory task. The cumulative hit rates for studied words and false alarm rates for novel items were calculated for each of the 10-point confidence ratings, ranging from 1–5 for “new” and 1–5 for “old”. These ROC functions were analyzed separately for negative and neutral words. The ROC curves (in Figure 4) were constructed as in previous recognition memory research (Leynes et al. 2019; Weidemann and Kahana 2016). According to the Dual Process Signal Detection (DPSD) model, Yonelinas and Parks (2007) have suggested in their review of ROCs and recognition memory that familiarity can create curved and symmetrical ROCs, while recollection can distort the ROCs to become more linear and asymmetrical. Therefore, the curvilinear ROC curves depicted in Figure 4 for our current data may signify familiarity-based recognition for negative and neutral words, rather than recollection-based processes. However, we need to proceed with caution. Although the figure presents potential trends in the impacts of various mechanisms, the underlying mechanisms still require testing.

### 3.3. Accuracies for Source Memory

For source memory, the data of both color retrieval and cue identification in each condition were analyzed using the conditional source identification measure (CSIM) rates. CSIM rates are the percentage of correctly identified source items in all the items that a participant identified as studied per condition (Bell and Buchner 2011; Nie and Li 2021; Schellhaas et al. 2020). There were two reasons for using the index CSIM rates. The first reason was that the retrieval of the current two sources, color and cue, was dependent upon whether an old word was correctly judged, and then the corresponding sources. However, it was unrelated to whether or not a new word was correctly discriminated. The second reason was that we used the sequential paradigm to test the source memory tasks, and responses for the source were only made for words that were believed to be old, neglecting the new words. Similar to item memory, because we had no predictions about the reaction times for source memory, we placed these data in the Supplementary Materials file.

The accuracies in color retrieval were also independently calculated, and the responses with high confidence (i.e., rated 4 or 5 on the confidence scale) were analyzed. The accuracy data of source memory were also analyzed separately using a repeated-measures ANOVA, with factors of expectation (expected, no expectation, and unexpected) and stimulus emotionality (negative and neutral). The accuracies (represented by CSIM rates) for source memory tasks, including overall color retrieval, high-confidence color retrieval, and cue identification, are summarized in Table 2.

The mean trial number between the two response sets of “overall responses” and “high-confidence responses” in color retrieval differed. Specifically, for expected negative words, the mean trial number of the overall response was 23, for the unexpected neutral words it was 24, for unexpected negative words it was 28, for expected neutral words it was 28, for negative words with no expectation it was 26, and for neutral words with no expectation it was 24. In addition, for expected negative words, the mean trial number of the high-confidence color retrieval was 17, for the unexpected neutral words it was 19, for unexpected negative words it was 20, for expected neutral words it was 19, for negative words with no expectation it was 18, and for neutral words with no expectation it was 21.

#### 3.3.1. Results for Color Retrieval

For color retrieval, separate repeated-measures ANOVAs were conducted between expectation and stimulus emotionality for the CSIM rates of overall responses and high-confidence responses.

**Figure 5:**
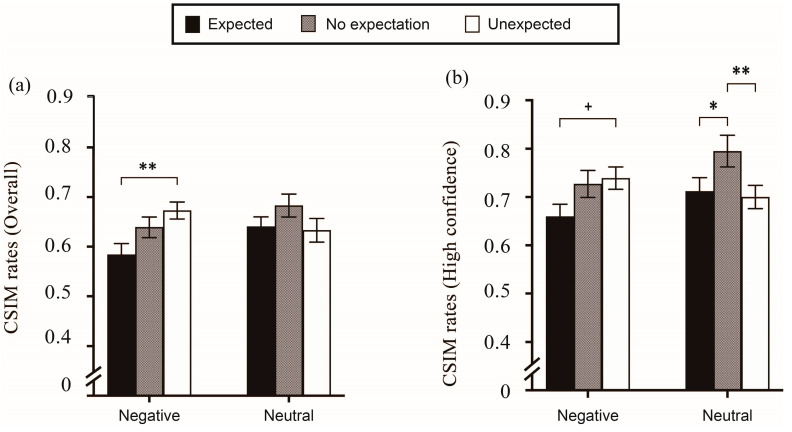
Performance in color-retrieval task (represented by CSIM rates) of the negative and neutral words in the expected, no-expectation, and unexpected conditions for (**a**) overall responses and (**b**) high-confidence responses. Bars represented the standard deviations (SDs). ^+^ *p* < 0.1, * *p* < 0.05, ** *p* < 0.01.

We tested the sphericity before the ANOVA. Within the overall responses, Mauchly’s test of sphericity revealed that the effect of expectation showed no indication of sphericity violation *(χ*^2^(2) = 0.276 *p* = 0.871), so the assumption of sphericity for the interaction was violated (*χ*^2^(2)= 8.127 *p* = 0.017, *ε* = 0.780). Therefore, the Greenhouse–Geisser correction was conducted on the effect of the interaction. The ANOVA revealed a significant main effect of expectation (*F*(2,58) = 7.148, *p* = 0.002, $\eta_{p}^{2}$ = 0.198), as shown in Figure 5a. The performance in the color-retrieval task for the expected words was worse than that for unexpected words (*t*(29) = −2.956, *p* = 0.014, Cohen’s *d* = −0.540) and worse than the words with no expectation as well (*t*(29) = −3.520, *p* = 0.003, Cohen’s *d* = −0.643). However, there was no apparent difference between the no-expectation and unexpected words (*p* = 1.000). 

Although there was no significant main effect of stimulus emotionality on the overall responses (*F*(1,29) = 2.740, *p* = 0.109, $\eta_{p}^{2}$ = 0.086), the expectation and stimulus emotionality interaction was significant, where the Greenhouse–Geisser correction was applied due to a sphericity violation (corrected *F*(2,46) = 4.915, *p*_corrected_ = 0.017, $\eta_{p}^{2}$ = 0.145). This suggests the distinct influence of expectation on the task of color retrieval for both the negative and neutral words. The post hoc comparisons revealed that only for the negative words, memory for the colors of expected stimuli was worse than that of unexpected stimuli (*t*(29) = −4.101, *p* = 0.001, MD = −0.090, 95% CI [−0.155, 0.024]). Regarding the overall responses, we confirmed not only the assumption of the main effect of expectation on the color-retrieval task but also its interaction with stimulus emotionality. However, no main effect of stimulus emotionality was found.

Another repeated-measures ANOVA was conducted for the high-confidence results. It should be mentioned that during the calculation of the high-confidence responses, the data of another three participants were excluded (i.e., 27 remaining) because few trials in their responses were made with high confidence. Therefore, the corresponding data were not relevant to the analysis. Mauchly’s test of sphericity observed no violation of the assumption of sphericity, neither for the effect of expectation (*χ*^2^(2) = 1.027, *p* = 0.598), nor for the interaction (*χ*^2^(2) = 0.575, *p* = 0.750). As a result, no correction was needed. We also found a prominent effect of expectation (*F*(2,52) =9.798, *p* < 0.001, $\eta_{p}^{2}$ = 0.274). Performance for the color retrieval of the expected words was worse than for the no-expectation words (*t*(26) = −4.418, *p* < 0.001, Cohen’s *d* = −0.850), and the unexpected words were slightly worse when compared with the no-expectation words (*t*(26) = −2.454, *p* = 0.053, Cohen’s *d* = −0.472). The patterns are depicted in Figure 5b. It seems that the data pattern in the color-retrieval task varied slightly between the overall responses and high-confidence responses.

There was also an interaction (*F*(2,52) = 3.907, *p* = 0.026, $\eta_{p}^{2}$ = 0.131), but no significant effect of stimulus emotionality (*F*(1,26) = 1.743, *p* = 0.198, $\eta_{p}^{2}$ = 0.063). Follow-up comparisons for the interaction showed that for the neutral words, there was a memory advantage of color retrieval in the no-expectation condition over the expected condition (*t*(26) = −3.128, *p* = 0.034, MD = −0.083, 95% CI [−0.163, −0.003]) and an advantage over the unexpected condition (*t*(26) = −3.563, *p* = 0.008, MD = −0.095, 95% CI [−0.175, −0.015]). For the negative words, only unexpected words were found to be worse than expected words, with a marginally significant difference (*t*(26) = −2.947, *p* = 0.060, MD = −0.078, 95% CI [−0.158, 0.002]). Similarly, in the high-confidence responses, we confirmed the assumption of the main effect of expectation on the color-retrieval task and its interaction with stimulus emotionality. However, no main effect of stimulus emotionality was detected.

#### 3.3.2. Results for Cue Identification

As with color retrieval, accuracies were calculated using the CSIM rates in the cue-identification task and were also submitted to the same ANOVA of expectation and stimulus emotionality. Before the ANOVA, we tested the sphericity. Mauchly’s test of sphericity did not yield any possibilities of sphericity violation, *χ*^2^(2) = 0.361, *p* = 0.835 for the effect of expectation, or *χ*^2^(2) = 4.806, *p* = 0.090 for the interaction. As a result, no correction was needed. The ANOVA revealed a main effect of expectation, *F*(2,58) = 43.827, *p* < 0.001, $\eta_{p}^{2}$ = 0.602. The CSIM rates of cue identification of words in the expected condition were much higher than those of words in both conditions of unexpected, *t*(29) = 8.716, *p* < 0.001, Cohen’s *d* = 1.591, and no expectation, *t*(29) = 7.318, *p* < 0.001, Cohen’s *d* = 1.336. The main effect of stimulus emotionality was not significant, *F*(1,29) = 1.860, *p* = 0.183, $\eta_{p}^{2}$ = 0.060.

**Figure 6:**
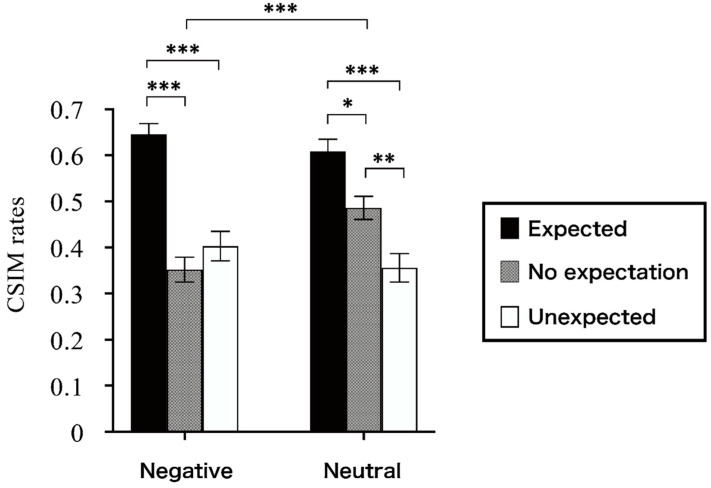
Performance in cue-identification task (represented by CSIM rates) of negative and neutral words in the expected, no-expectation, and unexpected conditions for overall responses. Bars represent the standard deviations (SDs). * *p* < 0.05, ** *p* < 0.01, *** *p* < 0.001.

In addition, the interaction of expectation and stimulus emotionality reached statistical significance (*F*(2,58) = 11.811, *p* < 0.001, $\eta_{p}^{2}$ = 0.289). The post hoc comparisons for this interaction revealed that a memory advantage for the cue identification of expected information was indiscriminately shown for negative words; expected > unexpected: *t*(29) = 6.811, *p* < 0.001, MD = 0.243, 95% CI [0.132, 0.354]; expected > no expectation: *t*(29) = 8.317, *p* < 0.001, MD = 0.294, 95% CI [0.205, 0.383]. In the neutral words, expected > unexpected: *t*(29) = 7.158, *p* < 0.001, MD = 0.253, 95% CI [0.175, 0.331]; expected > no expectation: *t*(29) = 3.471, *p* = 0.011, MD = 0.123, 95% CI [0.017, 0.229]. These patterns are presented in Figure 6. For the neutral words, the CSIM rates of the cue-identification task in the unexpected condition were worse than those in the no-expectation condition (*t*(29) = −3.687, *p* = 0.005, MD = −0.130, 95% CI [−0.236, −0.024]). Meanwhile, when the words were studied in the condition of no expectation, the cue-identification CSIM rates for negative words were worse than that for neutral words (*t*(29) = −4.953, *p* < 0.001, MD = −0.134, 95% CI [−0.216, −0.052]). 

In summary, in the CSIM rates of the cue-identification task, there was no reliable main effect of stimulus emotionality. However, it was confirmed that the assumption of a main effect of expectation, as well as the interaction between expectation and stimulus emotionality, was held.

We conducted a two-hour experiment to determine if the participants’ behavior remained consistent across different blocks. Specifically, we wanted to ascertain whether the pattern of expected and unexpected words over the words of no expectation persisted throughout all of the blocks. To examine this pattern, we analyzed the data from the beginning and end blocks. We conducted a supplemental analysis of the first two and the last two blocks. It was found that the first two blocks exhibited a similar pattern of overall responses, including the main effect of expectation and the interaction of expectation with stimulus emotionality. However, the last two blocks did not show the same interaction of these two variables, suggesting that the contribution of stimulus emotionality became weaker over time. Furthermore, the supplemental data revealed that the CSIM rates of the color-retrieval task were significantly lower in expected words than in no-expectation words in the last two blocks but not in the first two blocks. There was no interaction between expectation and stimulus emotionality. Thus, it was evident that the source memory of color retrieval behaved differently from the item memory task. For more detailed data, please refer to the Supplementary Materials file.

## 4. Discussion

The impact of pre-existing expectations on episodic memory tests has become a topic of growing interest. However, little attention has been given to the effects of experimentally induced expectations on subsequent memory performance. Examining both types of expectations is crucial for developing a thorough understanding of their respective influences on memory processing. Moreover, the majority of investigations have only focused on the two extreme ends of the continuum of expectation (i.e., expected and unexpected), leaving the middle ground largely unexplored. The current study, on the basis that information we received every day could be either expected or not, investigated how experimentally established expectations across the three levels (expected, no expectation, and unexpected) contributed to the retention of words. This included the two subtypes of episodic memory: item and source memory. Beyond that, we considered the factor of stimulus emotionality.

To explore the effects of our interest from a more thorough perspective, one rule-learning, one study, and three memory tasks, including item memory, color retrieval, and cue identification, were conducted. Our manipulation of experimentally established expectations in both the rule-learning and study phases was successful because the two phases were both reliably higher than chance. The accuracy of rule learning paved the way for the participants to generate the correct expectations in the subsequent study phase. During the study phase, the participants showed high accuracy in applying the correct rules to form appropriate expectations, or not to have any expectations at all. 

However, it is important to note that not all trials during the study phase followed the same rules as the trials generated during the rule-learning phase. As a result, the participants had to switch their response strategies and update their memory when faced with different rules in subsequent trials. The switch-and-update process allowed them to make relevant responses during subsequent tests. Our findings revealed that the impact of expectation on memory performance differed between negative and neutral words across the tests. The implications of the main findings and possible theoretical accounts concerning the findings are discussed below. Implications for future research directions are also provided.

### 4.1. Item Memory: A Standard “U-Shape” Is Shown for Neutral Words, but Is Distorted by Negative Emotionality

Expecting information appears to endow the subsequent item memory with a considerable advantage, regardless of the expectation conditions. As we predicted, words in the two expecting conditions were better recognized than those that were studied without an expectation, rendering a “U-shape” pattern for the impact of expectation on item memory. This “U-shape” finding replicates the result pattern observed in Experiment 1 and Experiment 3 of Greve et al. (2019) and was consistent with other investigations that found both expected and unexpected information could enhance the recall of the central event (for instance, Rae Tuckey and Brewer 2003). The SLIMM model (van Kesteren et al. 2012) outlines that anticipated information, which aligns with the schema, is handled via the schema-sensitive area, the mPFC, acting as a direct route for schema-congruent events. Nevertheless, unexpected information is conveyed through a distinct process, where the MTL, housing the hippocampus, plays a central role. It is noteworthy that any information falling between completely expected and unexpected can activate either the mPFC or MTL entirely, as the two processes suppress each other. 

The difference in processing schema-congruent and schema-incongruent events has not only been supported by the theoretical framework but also by empirical findings. The findings of several functional magnetic resonance imaging (fMRI) investigations found that the mPFC and MTL were in charge of processing expected and unexpected information, respectively (e.g., in Bonasia et al. 2018; van Kesteren et al. 2013). In addition, a study that applied an eye-tracking technique also found a difference, in that unexpected stimuli showed an increase in terms of both fixation and pupil dilation (Kafkas and Montaldi 2015). Therefore, both expected and unexpected information proved the better retention of item memory than the stimuli in the condition of expecting nothing in the current study, which suggested that both the first two types of information were processed well and could provide behavioral evidence for the validity of the SLIMM model.

Despite a “U-shape” pattern of expected and unexpected words appearing over words with no expectations, item memory performance for both expected and unexpected events did not show any discrepancies. This outcome is in line with the findings of Greve et al. (2019). However, in other investigations that applied pre-existing expectation, a more prominent pattern that expected information was better remembered than unexpected information was found (e.g., in van Kesteren et al. 2010). The current study found a trend towards an advantage for expected words; the distinct patterns suggested a difference between pre-existing and experimentally established expectation. Pre-existing expectation evoked a stronger sense of expectation, while experimentally induced expectation mitigated the feeling of expected and unexpected words. Expectation formed by pre-existing schema is crucial to human survival because the capability may help people deal with dangers in daily life. However, our surroundings can change rapidly, and unexpected situations can occur at any time. Therefore, we must rely on existing schemata to make decisions quickly.

It is worth noting that the “U-shape” pattern of both expected and unexpected words over the words of no expectation remained unchanged only in the case of neutral words, as previously observed by Greve et al. (2019) when using non-emotional stimuli. However, in the case of negative words, the “U-shape” pattern was altered as only the expected half of the curve existed. This outcome implied that only expected negative words were better remembered than those with no expectation, indicating a reliable interaction between expectation and stimulus emotionality. The interaction could be associated with similar processing areas of unexpected and negative information. As aforementioned, negative events have been confirmed to activate the temporal lobes, which is consistent with the information processing of unexpected conditions that relies heavily on the medial temporal lobes (Bonasia et al. 2018; Mickley and Kensinger 2009; van Kesteren et al. 2013). The processing routes of negative words could overlap with those of unexpected events. Therefore, stimulus emotionality could reduce the advantage of the unexpected condition on memory performance.

We also found that expectation could affect the influence of stimulus emotionality. Despite an overall EEM effect, the memory advantage of negative words over neutral words was only found in the condition when participants were not expecting anything. This outcome is similar to the results of a majority of investigations that did not introduce the factor of expectation to study the EEM effect (Minor and Herzmann 2019; Santaniello et al. 2018; Ventura-Bort et al. 2020; Weymar et al. 2013). This finding suggests that emotional stimuli can automatically capture more attention resources during encoding, leading them to be better stored with elaborative rehearsal that also facilitates subsequent retrieval, regardless of whether a word is expected.

However, when both emotional and non-emotional information were studied in terms of expectation, whether the information was expected or unexpected, no significant difference was observed in subsequent item memory. Lin and Liang (2020) similarly found that the item memory of negative and neutral items was similar when they were expected. However, in their study, negative items were worse recognized than neutral items in the unexpected condition, which is inconsistent with our results. This divergence between studies might be caused by the different retrieval tasks. The participants in our current study were aware that the studied items would be subsequently tested, whereas Lin and Liang (2020) applied a surprise item-recognition task. 

Second, the observed divergence in results may also be attributable to the differences in design between our study and that of Lin and Liang (2020). While their study only featured the item memory task, we incorporated three tasks into ours: item memory, color retrieval, and cue identification. With multiple tasks to attend to, the participants must allocate their cognitive resources and shift their response strategies while also updating their memory, particularly during the study phase. Specifically, the allocation of cognitive resources and response strategy switching may differ between neutral and negative words with different expectations. In neutral words, there is no differentiation between expected and unexpected situations in cognitive processing. In contrast, for negative words, more cognitive resources are allocated to expected situations before being allocated to unexpected words, with fewer resources allocated to situations where there is no expectation. 

A third perspective to explain the distinction between neutral and negative words is through reaction times. The data analysis regarding reaction times demonstrated that negative words resulted in faster item memory compared to neutral words (refer to the Supplementary Materials file for more information), potentially explaining the trade-off responses observed with negative words. A fourth explanation for the differences between studies might be the feature of the stimuli—pictures in Lin and Liang (2020) and words in our current case—because pictures need two specific phases: object identification and word production (Nie et al. 2021b). The fifth alternative might be that the negative emotionality itself could be a means of experimentally creating a violation of expectations. 

Furthermore, our supplemental analysis revealed that the pattern described above was not uniform in the initial and final blocks. Participants were able to adhere to the instructions of the task in the study phase even in the later blocks, resulting in their influence of expectation on memory being consistent throughout the study. However, one possibility is that the impact of negative emotion on memory performance decreased over time due to an increase in memory load, causing the memory of different valences to be difficult to distinguish.

Additionally, the curvilinear shape of the ROC curves, which depicts the discriminability of item memory, suggested that item memory processing was more dependent on familiarity rather than recollection, which coincides with the dual-process models (Leynes et al. 2019; Yonelinas and Parks 2007). 

### 4.2. Source Memory of Colors: No Expectation Leads to Better Retrieval of Contextual Details—A Reversed “U-Shape”, and No Interaction between Expectation and Stimulus Emotionality

For the sake of remembering colors, when we compared the CSIM rates of the overall responses and high-confidence responses, we found that despite several slight differences, they were generally identical in terms of trends. Regarding the effect of expectation, there was a clear reduction in CSIM rates for expected words related to colors, and a significant advantage for color retrieval was observed in the condition of no expectation. This is an exact reversal of the “U-shape” of item memory. However, for neutral words, the source memory of color retrieval was harmed in both expected and unexpected conditions; we observed no considerable difference between them. For negative words, only the expected words were remembered worse, and surprisingly, unexpected words were protected from memory loss and even gained superiority over their expected counterparts.

This reversed “U-shape” for neutral stimuli was found to be inconsistent with the patterns observed in Greve et al. (2019), where non-emotional stimuli were employed, and a graded pattern that was more similar to the negative stimuli was revealed. This inconsistency could be attributed to the differences in experimental manipulations. Specifically, in Greve et al.’s study, participants were instructed to simultaneously learn the rules and encode the events, whereas we split the tasks into two separate phases. Additionally, the difference in material types may have contributed to the observed discrepancy, as Greve et al. (2019) applied pictures as stimuli while we adopted words. Future investigations could explore these methodological differences thoroughly.

Interestingly, there was not any indication as to whether the colors of emotional or non-emotional stimuli were better remembered. This is consistent with our recent investigation on emotional words, where behavioral results showed no difference in retrieving the colors for negative and neutral words (Zhou et al. 2020). Similar findings have also been observed in other studies (Davidson et al. 2006; Earles et al. 2016; Pereira et al. 2019), of which Davidson et al. (2006) applied the emotionally valenced words from the ANEW as materials. Such a lack of difference was also supported by the Arousal-Biased Competition (ABC) theory raised by Mather and Sutherland (2011). The theory claims that stimulus emotionality can increase the competitive advantage of high-priority stimuli while impairing that of low-priority stimuli. Furthermore, the priority is mainly determined by both the significant perceptual features of the stimuli and the priority processing instruction relevant to the targets (Clewett et al. 2017; Mather and Sutherland 2011). In our current study, the participants were explicitly aware of the subsequent tests on colors. As a result, a top-down process was equally applied to the colors related to both negative and neutral words.

As mentioned above, when we compared the accuracy of item memory and source memory for colors, clearly distinct patterns were found. The item memory advantage for expected events, despite being inappreciable compared with unexpected events, did not extend to color retrieval, even showing an obvious disadvantage. This outcome was consistent with the overall responses and showed that color retrieval for source memory had differing processes compared to the item memory task. The results depicted that there were processing differences in the mechanisms, rather than just differences in memory strength, between item and source memory, specifically a subtype of source memory. These findings provide additional evidence for dual-process models of memory (Cooper et al. 2017; Leynes et al. 2017; Nie et al. 2019b; Ye et al. 2019), rather than models that claim a single process during the test (Hayes et al. 2017; Slotnick and Dodson 2005).

### 4.3. Source Memory of Cues: An Absolute Advantage for Expected Words, Supporting the Facilitation of Goal-Relevant Information, but No Interaction between Expectation and Stimulus Emotionality

Source memory is the memory for the contextual information of an event (Bell et al. 2016; Li and Nie 2021; Nie et al. 2019b). As such, in the current study, the memory of accompanied cues could also be categorized as source memory. However, as presented in Figure 4 and Figure 5, the performance in color-retrieval and cue-identification tasks were not parallel despite both being measures of source memory. For instance, as we discussed above that there was no recall difference between negative and neutral words for color retrieval, such a silent impact of stimulus emotionality was not found in the memory performance on the cues (i.e., cue identification). We found that when the information was studied without any expectation, there was an impairment of stimulus emotionality on memory for preceding cues. Specifically, the cues that preceded negative words were remembered worse than those of neutral words. In addition, the memory performance of the cues across the three conditions of expectation had little in common with that of the colors.

Although both tasks, color retrieval and cue identification, can be classified as source memory tests, the current findings indicate that they cannot be analyzed from the same standpoint. This suggests that distinct criteria might exist for categorizing these tests as distinct subtypes of source memory. The most easily approachable subtypes of source information were defined by their relationship with the core items, namely intrinsic and extrinsic sources (Mao et al. 2015; Zhou et al. 2020). Intrinsic sources are contextual information within an item. For instance, if a notebook is a target, then the color of the notebook would be an intrinsic source. In contrast, extrinsic sources are not directly related to the items but are contexts associated with them, for example, the color of the desk on which the notebook is placed. Based on this classification, the color-retrieval task in our current study examined the memory of intrinsic sources, while the cues were a type of extrinsic source. Thus, the latter task of cue identification might be more resource-consuming than the former task of color retrieval. Mao et al. (2015) have found that intrinsic sources of emotional information can be enhanced in memory, while the memory of extrinsic sources is impaired when the items carry emotionality.

On the one hand, the distinction between extrinsic and intrinsic sources was to some extent convincing, as in our current study, the preceding cues (i.e., extrinsic sources) were better remembered for neutral words when compared with the negative words, in the condition of no expectation. On the other hand, this distinction was to some degree untenable because the memory for colors showed no difference between negative and neutral words. Therefore, we advise not to simply categorize contextual information as intrinsic or extrinsic. Alternatively, another classification disregards the integral attribution of contextual information and instead focuses on the relationship with the encoding goal. For example, Sweegers et al. (2015) found that if the schema is the main focus for achieving the goal, then the goal-related input is facilitated. Applying this classification to our current study, we encoded the cues and words with a semantic expectation, serving as the goal-related input. Meanwhile, the colors of the words were intended to be perceived as goal-irrelevant. 

In accordance with Sweegers et al.’s theories, there was an improvement in item memory performance for expected information, but impairment in the memory of the displayed colors, which were irrelevant to the study task. Additionally, recalling the cue using its subsequently presented words could be compared to the backwards-cued recall task in Frank et al. (2018). In their study, participants were asked to recall the previous event of a target, which was presented sequentially during the study. Similarly, they found that memory performance was superior for congruent over incongruent and unrelated events, which aligns with our current findings.

However, no previous investigations have observed a reduction in memory for cues in unexpected information for neutral words. We assume that this phenomenon could also be caused by the stimulus types used in our study. Other studies that investigated the influence of expectation on source memory required to remember the “seeing” targets, such as pictures and scenes in Frank et al. (2018) and Greve et al. (2019) and faces in Sweegers et al. (2015). In contrast, our current study focused on the “reading” targets, specifically verbal words.

In summary, we observed an absolute advantage of expected information in the memory of preceding cues. This resulted in different patterns of recall for negative and neutral words, where the memory of the cue for unexpected negative words was not reduced as sharply as for neutral words. This indicates the joint contribution of expectation and stimulus emotionality on the source memory for cues. In addition to the various patterns in different types of sources, the current study also found a noticeable divergence between item and source memory. This finding is consistent with other investigations conducted over the years (Ehrenberg and Klauer 2005; Nie et al. 2019a).

### 4.4. Limitations and Future Directions

The current study revealed several impacts of expectation and stimulus emotionality on both item and source memory. However, some limitations in our current experiment were exposed.

First, regarding the valence and arousal of the affective words used in our study, we were unable to control the arousal level due to insufficient word options for our study and test materials. For the same reason, we only included negative and neutral words, omitting positive valence. As a result, we were unable to determine whether the emotion-related impacts observed in the current study were attributed to arousal, valence, or both. 

The relationship between valence and arousal has always been a matter of controversy, which has been investigated by a large group of researchers. Several theoretical models have been developed to explain how the memory of arousing events differs from other events (for a comprehensive review, see Bowen et al. 2018). From the results obtained, supporters of the “trade-off” theory argue although that arousing events can improve the memory of essential information or the general idea, but at the expense of other details (Adolphs et al. 2005). The ABC theory assumes that the EEM effect can occur regardless of the central/peripheral distinction and is closely related to arousal. Therefore, even contextual information can be better remembered if it is arousing (Mather and Sutherland 2011). There are also investigations on how valence itself could affect memory. Among them, a majority of studies show the superiority of item memory for negatively valenced information, as well as a relative impairment regarding their source memory. This impairment can either be equal to or inferior to the neutral information (Bookbinder and Brainerd 2017; Kamp et al. 2015; Mickley et al. 2016; Mneimne et al. 2010). Besides, a “NEVER” theory that proposes the memory advantage of negative valence has been devised by Bowen et al. (2018). Therefore, to provide supportive evidence for either theoretical model in the future, it would be wise to consider both valence and arousal levels.

Second, besides the level of “no expectation” in our current study, other levels of expectation should also be considered. In van Kesteren et al.’s (2013) study, they manipulated an intermediate level of “less congruent” in addition to the expected and unexpected levels of expectation. This manipulation was similar to the “low typicality” manipulation in Höltje et al. (2019). In contrast to our findings and those of Greve et al. (2019), which showed a “U-shape” pattern where both expected and unexpected events were better remembered than events with no expectation, van Kesteren et al.’s (2013) and Höltje et al.’s (2019) investigations found a graded pattern where expected information was significantly more memorable than unexpected information, which was the least memorable. These distinct patterns inspire future studies to consider all the levels of expectation and to investigate whether a level of “less incongruent” also exists and how it differs from the “less congruent” level. Additionally, it is highly recommended for future studies to explore expectation for both items and sources. We suspect different patterns may be observed when the expectation is source-related rather than item-related.

Third, as we demonstrated by our behavioral results, we suspected that expectation and stimulus emotionality were not independent factors influencing memory, but rather had common processes. Neurological research suggests that the memory of positive and neutral information is associated with frontal activity, whereas negative information activates more temporal areas (Mickley and Kensinger 2009). Combined with the aforementioned SLIMM model, which claims that mPFC is more activated for expected information and MTL for unexpected events (see van Kesteren et al. 2012 for a review), this assumption appears to be well-supported. However, further investigation is needed to determine the extent to which these factors overlap in the memory process and their common processes. We admit that our current study is a behavior experiment and not a neural experiment, and this cannot precisely offer evidence either to support or rebut the SLIMM. Future studies are strongly encouraged to adopt the high-temporal-resolution event-related potential (ERP) technique and high-spatial-resolution fMRI technique to characterize brain activity when expectation and stimulus emotionality are considered simultaneously.

Fourth, it is significant to consider the choice of paradigm when investigating source memory. Based on current understanding, three paradigms can be utilized, namely the sequential paradigm, the exclusion paradigm, and the three-key paradigm. In our study, we employed the sequential paradigm, in which the responses for item and source memory were made consecutively; item memory was tested before source memory. In the exclusion paradigm, participants are required to differentiate between items learned from source A and other items. In the three-key paradigm, participants must provide three separate responses, one response for items studied from source A, one response for items from source B, and another response for new items. It is possible to compare the two memory tasks as their responses are simultaneous.

Fifth, the feature of stimuli might be considered. As mentioned, pictures need two specific phases—object identification and word production (Nie et al. 2021b)—which are unnecessary in words. These specific phases may cause the expectation generation to be different between pictures and words of negative valence. We expected to observe a “U-shape” pattern in both expected and unexpected words when compared to words with no expectation in our current item memory task. Furthermore, we will investigate the possible interplay between expectation and stimulus emotionality when utilizing pictures as stimuli. 

Last, it should be noted that we had two variables—expectation and stimulus emotionality, resulting in 6 measurements. Thus, when conducting the a priori test for sample size, we put 6 into the G*Power software and obtained a reasonable participant number of 26. One might be wondering whether the current findings would be upheld if the measurement number was decreased. A follow-up choice might be to increase the sample size, such as 52, to increase power.

## 5. Conclusions

Although there is increasing research on the influence of expectation on subsequent memory, the current study manipulated experimentally established expectation. This manipulation included a condition with no expectation, in addition to the expected and unexpected conditions. The results revealed that expectation and stimulus emotionality can converge to affect both item and source memory. In item memory, we discovered a standard “U-shape” relationship, where both expected and unexpected neutral words were better remembered than the no-expectation words. This outcome provides evidence of the validity of the SLIMM theory. Different patterns were observed for source memory, where expected events lacked a heightened memory for contextual details such as colors but resulted in an outstanding performance in recalling cues. This suggests that the influence of expectation could differ between item and source memory, supporting dual-process models over single-process models. Furthermore, this study showed that the impact of expectation acted differently when it came to memory for different types of source information, highlighting the need for accurate classification theories for source memory.

## Figures and Tables

**Table 1:** Mean accuracy (hits and Prs) and d’ across the conditions in item memory, separately for overall responses and high-confidence responses. The corresponding standard deviations (SDs) are included in parentheses. The “Prs” is short for “Pr scores” and “No exp.” is short for “No expectation”. “Overall responses” and “High-confidence responses”, respectively, indicate the “Overall responses on item memory” and “High-confidence responses on item memory”, and d’ is short of “Z_hits_—Z_false alarms_”.

	Negative Words			Neutral Words		
	Expected	No exp.	Unexpected	Expected	No exp.	Unexpected
Overall responses						
Hits	0.86 (0.07)	0.79 (0.09)	0.83 (0.08)	0.85 (0.06)	0.74 (0.11)	0.83 (0.08)
Prs	0.72 (0.12)	0.66 (0.12)	0.70 (0.13)	0.67 (0.15)	0.56 (0.19)	0.64 (0.17)
d’	−0.022 (1.35)	−0.052 (1.17)	−0.041 (1.33)	0.015 (1.54)	−0.005 (1.54)	0.00035 (1.44)
High-confidence responses						
Hits	0.93 (0.05)	0.88 (0.10)	0.92 (0.07)	0.93 (0.07)	0.82 (0.12)	0.89 (0.10)
Prs	0.82 (0.12)	0.77 (0.14)	0.81 (0.13)	0.81 (0.15)	0.70 (0.19)	0.77 (0.16)
d’	−0.011 (1.21)	−0.013 (1.41)	0.002 (1.41)	0.039 (1.51)	0.022 (1.62)	0.024 (1.43)

**Table 2:** Mean accuracy (CSIM rates) across the conditions in source memory (including overall color retrieval, high-confidence color retrieval, and cue identification). The corresponding standard deviations (SDs) are included in parentheses. “No exp.” is short for “No expectation”, “CSIM” is short for “Conditional Source Identification Measure”. “Overall” and “high-confidence”, respectively, indicate the “Overall responses on color-retrieval task” and “High-confidence responses on color-retrieval task”.

Negative Words			Neutral Words		
Expected	No exp.	Unexpected	Expected	No exp.	Unexpected
Color retrieval (overall)					
0.58 (0.12)	0.64 (0.11)	0.67 (0.10)	0.64 (0.11)	0.68 (0.13)	0.63 (0.13)
Color retrieval (high confidence)					
0.66 (0.13)	0.73 (0.14)	0.74 (0.12)	0.71 (0.14)	0.79 (0.17)	0.70 (0.13)
Cue identification					
0.65 (0.13)	0.35 (0.15)	0.40 (0.17)	0.61 (0.14)	0.49 (0.14)	0.37 (0.17)

## Data Availability

The current datasets generated and/or analyzed are publicly available in an open data repository.

## Supplementary Materials

The following supporting information can be downloaded at: https://www.mdpi.com/article/10.3390/jintelligence11070130/s1, Data S1: Results comparison between the first two and the last two blocks, Data S2: Reaction times.
