# Supplementary material for: Does Expecting Matter? The Impact of Experimentally Established Expectations on Subsequent Memory Retrieval of Emotional Words
Source: J Intell. 2023 Jul 1;11(7):130. doi: 10.3390/jintelligence11070130 (PMC10381812; doi:10.3390/jintelligence11070130)
Supplement: Supplementary file 1 [file jintelligence-11-00130-s001.zip › jintelligence-2325702-supplementary.pdf]

**Supplemental data**

**S1. Results comparison between the first two and the last two blocks**

**During the study phase**, the identifications for whether the target words were expected, with no expectation, or unexpected, were better in the first two blocks ($M$ = 0.964, $SD$ = 0.046) than in the last two blocks ($M$ = 0.879, $SD$ = 0.080), indicating that the participants reduced their identifications when times went by. Nonetheless, both the data were prominently higher when compared to the chance level of 0.333, $t(29)$ = 57.989, $p$ < 0.001, Cohen's $d$ = 13.668, and $t(29)$ = 28.024, $p$ < 0.001, Cohen's $d$ = 6.797. This suggested that our participants could use the rules they learned during the rule learning phase to guide them to make responses during the study phase.

**Besides the study phase, we also made data analyses for the three test phases**. To make the data simple, we only analyzed the overall response condition, i.e., hit rates and Prs for the item memory task, and the CSIMs for both the color retrieval and the cue identification tasks.

**First, for the first two blocks of the item memory**, the hit rates were submitted to the two-way repeated-measures ANOVA of expectation by stimulus emotionality. The ANOVA found a reliable main effect of expectation, $F(2,58)$ = 13.078, $p$ < 0.001, $\eta_p^2$ = 0.483. The post hoc test found higher hit rates for targets with expectations than those without any expectation: expected words were performed better than no expectation words, $t(29)$ = 2.400, $p$ = 0.020, Cohen's $d$ = 0.618; unexpected words also showed a memory advantage over the no expectation words, $t(29)$ = 3.880, $p$ < 0.001, Cohen's $d$ = 1.008, while no difference was found between expected and unexpected words of item memory, $t(29)$ = 1.547, $p$ = 0.127, Cohen's $d$ = 0.402. There was also a significant interaction between expectation and stimulus emotionality, $F(2,58)$ = 3.546,

$p = 0.042$, $\eta_p^2 = 0.202$. A simple effect test for this two-way interaction found that the role of expectation was modulated by the factor of stimulus emotionality, showing that for the no-expectation condition, the hit rates were much higher for negative words than for neutral words, revealing a reliable EEM effect in the hit rates of the first two blocks of the item memory task.

**Regarding the last two blocks**, the same ANOVA for the hit rates only revealed a reliable main effect of expectation, $F(2,58) = 14.082$, $p < 0.001$, $\eta_p^2 = 0.501$. The post hoc test found much higher hit rates for targets with expectations than those without any expectation: expected words were performed better than no expectation words, $t(29) = 4.499$, $p < 0.001$, Cohen's $d = 1.170$; unexpected words also showed a memory advantage over the no expectation words, $t(29) = 3.181$, $p = 0.002$, Cohen's $d = 0.828$, while no difference was found between expected and unexpected words of item memory, $t(29) = 1.175$, $p = 0.245$, Cohen's $d = 0.305$. This showed that regarding the main effect of expectation, the same pattern was shown between the first two blocks and the last two blocks, that is, for the hit rates, both expected and unexpected words acted much higher than no expectation; while the pattern was only modulated by stimulus emotionality in the first two blocks but not the last two blocks.

**For the Prs of the first two blocks**, the two-way repeated-measures ANOVA revealed both the main effects of expectation and stimulus emotionality, the effect for the expectation was $F(2,58) = 13.078$, $p < 0.001$, $\eta_p^2 = 0.483$. The post hoc test for the factor of expectation found that the Prs for expected words was performed better than no expectation words, $t(29) = 2.337$, $p = 0.023$, Cohen's $d = 0.674$; unexpected words did not show a memory advantage over the no expectation words, $t(29) = 1.437$, $p = 0.156$, Cohen's $d = 0.402$, and no difference was found between expected and unexpected words of item memory, $t(29) = 0.868$, $p = 0.389$, Cohen's $d = 0.229$. The

main effect for stimulus emotionality was $F(1,29) = 10.376$, $p = 0.006$, $\eta_p^2 = 0.426$, the post hoc test found that the Prs were larger in negative words than in neutral words, showing a reliable EEM effect in the Prs of the item memory. There was also the reliable two-way interaction $F(2,58) = 3.546$, $p = 0.042$, $\eta_p^2 = 0.202$. The simple effect tests for this interaction found that both the expected and no expectation conditions revealed the reliable effect of stimulus emotionality, $F(1,29) = 15.86$, $p < 0.001$ and $F(1,29) = 10.17$, $p = 0.007$.

**For the Prs of the last two blocks**, we revealed the main effects of expectation and stimulus emotionality, $F(2,58) = 14.082$, $p < 0.001$, $\eta_p^2 = 0.501$ and $F(1,29) = 4.601$, $p = 0.050$, $\eta_p^2 = 0.247$. The post hoc test for the factor of expectation found no difference between expected and unexpected words in Prs of item memory, $t(29) = 0.522$, $p = 0.604$, Cohen's $d = 0.134$; unexpected words did not show a memory advantage over the no expectation words, $t(29) = 1.714$, $p = 0.092$, Cohen's $d = 0.442$; while expected words were performed better than no expectation words, $t(29) = 2.222$, $p = 0.030$, Cohen's $d = 0.391$. The post hoc test for the factor of stimulus emotionality found that negative words behaved better than neutral words, demonstrating a reliable EEM effect in Prs of the last two blocks. In sum, for the Prs, both the first two blocks and the last two blocks showed that the expected targets performed higher than no expectation ones, while that in the first two blocks was modulated by stimulus emotionality but not in the last two blocks.

**Second, for the CSIMs of the color retrieval of the first two blocks**, the ANOVA only revealed a marginally significant main effect of expectation, $F(2,58) = 2.968$, $p = 0.068$, $\eta_p^2 = 0.175$. Subsidiary analyses did not find the CSIMs difference between expected and no expectation conditions, $t(29) = 1.149$, $p = 0.255$, Cohen's $d = 0.352$, between unexpected and no expectation conditions, $t(29) = 0.401$, $p = 0.690$, Cohen's

*d* = 0.651, and also between expected and unexpected conditions, $t(29) = 1.636$, $p$ = 0.107, Cohen's $d = 0.120$.

**For the CSIMs of the color retrieval of the last two blocks**, the ANOVA revealed a significant main effect of expectation, $F(2,58) = 4.340$, $p = 0.023$, $\eta_p^2$ = 0.237, and the two-way interaction of expectation by stimulus emotionality, $F(2,58)$ = 4.943, $p = 0.015$, $\eta_p^2$ = 0.261. Post-hoc test found that the expected condition did not differ from the unexpected condition, $t(29) = 1.637$, $p = 0.107$, Cohen's $d = 0.423$, and the unexpected condition was similar to the no expectation condition, $t(29) = 0.822$, $p$ = 0.414, Cohen's $d$ = 0.213, but the expected condition was lower than the no expectation condition, $t(29) = 0.2.419$, $p = 0.019$, Cohen's $d = 0.628$. The simple effect test for the two-way interaction found that the role of the expectation was marginally modulated by the factor of stimulus emotionality in the three levels of expectation, $F(1,29) = 3.91$, $p = 0.068$, $F(1,29) = 3.73$, $p = 0.074$, and $F(1,29) = 3.61$, $p = 0.078$ for expected, unexpected, and no expectations, respectively. In sum, the CSIMs of the color retrieval task were much lower in expected words than in no-expectation ones in the last two blocks but not in the first two blocks.

**Third, for the CSIMs of the cue identification of the first two blocks**, there was a significant main effect of expectation, $F(2,58) = 5.703$, $p = 0.008$, $\eta_p^2$ = 0.289. Also, there was reliable two-way interaction of expectation by stimulus emotionality, $F(2,58)$ = 41.763, $p$ <0.001, $\eta_p^2$ = 0.749. Post-hoc test for the main effect of expectation found that expected targets did not differ from the unexpected ones, $t(29) = 0.163$, $p = 0.871$, Cohen's $d$ = 0.042; the unexpected condition was marginally different from the no expectation condition, $t(29) = 1.861$, $p = 0.068$, Cohen's $d = 0.480$, and the expected condition was marginally higher than the no expectation condition, $t(29) = 1.956$, $p$ = 0.055, Cohen's $d = 0.505$. Regarding the two-way interaction, the stimulus emotionality

could modulate all three levels of expectation, showing the expected, unexpected, and no expectation words all behaved better in negative valence than in neutral valence, $F(1,29) = 45.39, p < 0.001$, $F(1,29) = 34.31, p < 0.001$, and $F(1,29) = 19.55, p < 0.001$. For the CSIMs of cue identification of the first two blocks, the expected and unexpected words were both marginally more detrimental than the no-expectation conditions.

**For the CSIMs of the cue identification of the last two blocks**, there was only a significant main effect of expectation by stimulus emotionality, $F(2,58) = 5.703, p = 0.008$, $\eta_p^2 = 0.289$. A simple effect test for this two-way interaction found that the stimulus emotionality could modulate all three levels of expectation, showing the expected, unexpected, and no expectation words all behaved better in negative valence than in neutral valence, $F(1,29) = 42.55, p < 0.001$, $F(1,29) = 11.03, p = 0.005$, and $F(1,29) = 15.14, p = 0.002$.

## S2. Reaction times

### S2.1. Reaction times (RTs) in item memory

The RTs in the item memory task were also submitted to the same repeated-measures ANOVA of expectation by stimulus emotionality. Before this ANOVA, we tested the sphericity first. Across the overall responses, as there was no violation of sphericity, $\chi^2(2) = 1.026, p = 0.599$ for expectation and $\chi^2(2) = 0.231, p = 0.891$ for the interaction, no Greenhouse-Geisser correction was made. The ANOVA confirmed that there was a main effect of expectation, $F(2,58) = 3.806, p = 0.028$, $\eta_p^2 = 0.116$, and a main effect of stimulus emotionality, $F(1,29) = 4.511, p = 0.042$, $\eta_p^2 = 0.135$, but no significant interaction, $F(2,58) = 0.112, p = 0.894$, $\eta_p^2 = 0.004$. Post hoc comparisons revealed that words in the expected condition were identified faster than their counterparts in the no expectation condition, $t(29) = 2.727, p = 0.025$, Cohen's $d$ = -

0.498, no difference in speed was found between expected and unexpected conditions, $t(29) = -1.000$, $p = 0.964$, Cohen's $d = -0.183$, nor between unexpected and no expectation conditions, $t(29) = -1.727$, $p = 0.269$, Cohen's $d = -0.315$. Negative words responded slower than neutral ones, $t(29) = 2.124$, $p = 0.042$, Cohen's $d = 0.388$. However, when only those with high-confidence responses were considered, there was not any effect that reached statistical significance. There was no main effect of expectation, $F(2,46) = 0.889$, $p = 0.397$, $\eta_p^2 = 0.030$, no main effect of stimulus emotionality, $F(1,29) = 1.935$, $p = 0.175$, $\eta_p^2 = 0.063$, and no significant interaction, $F(2,58) = 0.661$, $p = 0.520$, $\eta_p^2 = 0.022$.

**S2.2. Reaction times in source memory**

In terms of the RTs in the color retrieval task, a similar repeated-measures ANOVA was conducted, with expectation and stimulus emotionality as within-subject factors. A main effect of expectation was obtained, $F(2,58) = 5.716$, $p = 0.005$, $\eta_p^2 = 0.165$, retrieving the colors for words of no expectation required less time than that for unexpected words, $t(29) = 3.259$, $p = 0.006$, Cohen's $d = 0.595$, and was marginally significantly faster than that for expected words, $t(29) = 2.408$, $p = 0.058$, Cohen's $d =$ 0.440. The effect of stimulus emotionality was significant as well, $F(1,29) = 23.584$, $p < 0.001$, $\eta_p^2 = 0.449$. Color retrieval for negative words responded slower than for neutral words, $t(29) = 4.856$, $p < 0.001$, Cohen's $d = 0.887$. In addition, there was a marginal significance in the expectation by stimulus emotionality interaction, $F(2,58) = 3.028$, $p = 0.056$, $\eta_p^2 = 0.095$, and the post hoc comparisons found that colors for negative words were retrieved slower than for neutral ones only when they were studied in the condition of no expectation, $t(29) = 4.576$, $p < 0.001$, MD = 69.909, 95% CI [23.763, 116.054].

Regarding the RTs in cue identification, both effects of expectation and interaction

showed sphericity violation in Mauchly's test of sphericity, $\chi^2(2) = 11.749$, $p = 0.003$, $\varepsilon = 0.745$, and $\chi^2(2) = 10.351$, $p = 0.006$, $\varepsilon = 0.764$, therefore, the effects were corrected through Greenhouse-Geisser method. The ANOVA did not produce any main effect of expectation, corrected $F(2,43) = 2.305$, $p_{\text{corrected}} = 0.125$, $\eta_p^2 = 0.074$, or any effect of stimulus emotionality, $F(1,29) = 1.540$, $p = 0.225$, $\eta_p^2 = 0.050$. However, there was a prominent interaction of expectation by stimulus emotionality, corrected $F(2,44) = 6.706$, $p_{\text{corrected}} = 0.006$, $\eta_p^2 = 0.188$. Post hoc comparisons showed that identifying the cues for negative words in the expected condition was faster than that in the no expectation condition, $t(29) = -3.753$, $p = 0.004$, MD = -101.360, 95% CI [-182.307, -20.412], and that when completing the cue identification task, negative words obtained a faster response than neutral words that were previously studied as expected, $t(29) = -3.496$, $p = 0.012$, MD = -104.568, 95% CI [-195.100, -14.035].
